# Non-accidental violence toward sport officials: a scoping review

**DOI:** 10.3389/fspor.2026.1765769

**Published:** 2026-03-25

**Authors:** Maria Luisa Fernanda Pereira Vargas, Tom Webb, Paul Gorczynski, Laura K. O’Keefe, David Hancock

**Affiliations:** 1 School of Human Kinetics & Recreation, Memorial University of Newfoundland, St. John’s, NL, Canada; 2 Faculty of Business and Law, Coventry University, Coventry, United Kingdom; 3 School of Human Sciences, University of Greenwich, London, United Kingdom

**Keywords:** abuse, judges, referees, synthesis, umpires

## Abstract

There is yet to be a focused review and synthesis on non-accidental violence toward sport officials despite a surge in academic inquiry into the topic over the last 25 years. As such, the aim of this review is to synthesize what is known about non-accidental violence toward sport officials. Articles were selected in March 2025. 32 articles were included in this scoping review with 13 countries and 7 sports represented. 43.7% of studies used qualitative methods, and only 4.1% of the total sample were women officials. Sport officials frequently experienced non-accidental violence, in particular verbal abuse from athletes, coaches, and spectators; organizations were identified as key contributors to the continuation of the problem. Non-accidental violence had serious consequences for sport officials including depression and attrition, particularly for those with limited organizational and/or social support. We recommend future researchers prioritize vulnerable and marginalized sport officiating groups and diversify methodological choices through employing in-depth qualitative methods, using robust demographic questionnaires, and expanding research questions.

## Introduction

Sport and violence have been described as synonymous (1). Violence (e.g., physical aggression between athletes) is an expected and even applauded part of many sports, as seen in contact and combat sports (2). This type of violence between athletes is well studied, with research and practice concerned with understanding and mitigating the problem and associated risks (3). However, the broader term of non-accidental violence—which is intentional harassment and abuse directed toward an individual in the form of psychological, physical, sexual, or neglect (4)—has often been left out of conversations about violence in sport until recently.

There has been an emerging body of work documenting non-accidental violence in sport (4). This work has focused on violence against athletes perpetrated by people in positions of authority such as coaches or members of an athletes’ entourage (5–7). Scholars have also focused on sport officials (e.g., referee, umpires, and judges), an oftentimes overlooked population within sport psychology literature (8). Increased academic inquiry, alongside a growing number of public accounts in the media, highlight that non-accidental violence directed toward sport officials is a growing cause for concern. These works illuminate the concerning psychological outcomes of experiencing non-accidental violence, such as anxiety and depression (9, 10). Further, non-accidental violence is contributing to an officiating crisis, with studies highlighting that up to 49% of officials quit due to abuse (11). Such consequences (e.g., attrition, more abuse) are heightened for those with perceived limited organizational support (12).

The culture of high-performance sport facilitates the experience of non-accidental violence toward sport officials (13), as it tends to operate as outcome-based, which places great value on measures of performance success (14). A win-at-all-costs ideology is widely adopted, reinforced within sporting cultures by athletes, coaches, spectators, and other staff operating within a sporting environment (15, 16). This ideology is an accepted driver of violence-supporting attitudes against sport officials. For example, sport officials have experienced verbal and physical abuse by coaches, athletes, and/or spectators when it is perceived that the officials have made an incorrect decision impacting a team's ability to win (17, 18). Knapton et al. (61) suggested spectators specifically used violence as a psychological, physical, and social mechanism to feel belonging and restore pride after experiencing sporting loss. Therefore, non-accidental violence and its impact in sport officiating populations is concerning, particularly because the very nature of high-performance sport might encourage abuse. As such, a synthesis of literature is timely and needed to understand mechanisms that reinforce abuse, its impact, and the gaps in the current literature to further support this population.

Taken together, the conceptual framework for this study proposes that situational factors (e.g., officiating decisions), cultural influences (e.g., win-at-all costs ideology), and individual factors (e.g., athlete, coach, or spectator perceptions of decisions), and organizational factors (e.g., disciplinary policies)—can influence non-accidental violence toward sport officials. Several reviews which synthesize findings related to non-accidental violence have been published. These works have focused on non-accidental violence toward all athletes (4, 62), parasport athletes (19), and women athletes (20). Beyond athletes, a review by Mojtahedi et al. (17) has also focused on abuse toward match officials (i.e., in team-based sports). However, there is yet to be a focused review and synthesis on non-accidental violence toward sport officials across all sports and levels of competition, despite a surge in academic inquiry into the topic in the last 25 years. As such, the aim of this review was to synthesize what is known about non-accidental violence toward sport officials.

## Methods

A scoping review was deemed the most appropriate review type to achieve our aims. Our aim was focused on synthesizing what is known about non-accidental violence toward sport officials through mapping and discussing findings rather than through assessing the appropriateness of articles (e.g., through quality criteria assessment) and meaningfulness of data, as would be seen in a systematic review. We also opted for a scoping review instead of a narrative review as the loose research criteria of a narrative review risks missing relevant articles and potential important insights (63).

This scoping review was conducted in accordance with PRISMA (21, 22) recommendations, and followed the framework set out by Arksey and O'Malley (23) which included the following steps: (a) identifying the research question; (b) identifying relevant studies; (c) selecting studies; (d) charting the data; and (e) summarizing and collating the data and reporting the results. The following sub-sections outline the first four stages, with stage five represented through the results section.

### Identifying the research question

Our research questions included:

1. How has non-accidental violence toward sport officials been researched?
2. What types of non-accidental violence do sport officials experience and to what extent?
3. Who enacts non-accidental violence toward sport officials, and which sub-groups are most at risk?
4. What is the impact of non-accidental violence?
5. How do sport officials cope, and what role do organizations play in supporting sport officials?

### Identifying relevant studies

The search for relevant articles took place in March 2025. To begin, the following databases were searched by two members of the research team (MPV, LO): APA PsycInfo, SPORTDiscus, Web of Science, and PubMed. The search criteria included three strands: (a) non-accidental violence (e.g., “abuse”, “harassment”); (b) sport; and (c) sport officials (e.g., “referee”, “judge”). See the Supplementary File for the full search terms and process.

We included articles that met the following criteria: (a) any type of non-accidental violence; (b) included any sport officiating population; (c) in any languages which the research team were literate in (English, Spanish, or Portuguese); and (d) peer-reviewed articles. These criteria were applied during the search stage. Articles which included a perspective other than sport officials were excluded [e.g., parents as studied by (64)]. To keep the review focused on non-accidental violence, any articles that mentioned a form of non-accidental violence but were not specifically focused on it (were instead focused on stress or mental health) were excluded [e.g., (65, 66)]. This decision was made to keep the review focused and to ensure comparability across studies. For the purpose of our study, all types of sport officials were included. No parameters were set on publication date with articles gathered from ‘earliest’ up until the search date (March 2025). Reviews, commentaries, consensus statements, grey literature, and dissertations were excluded. Reference lists of articles that met inclusion as well as review articles in the research area were also scanned to ensure other relevant articles were not missed [e.g., (9, 17, 24)].

### Selecting studies

**Figure 1:**
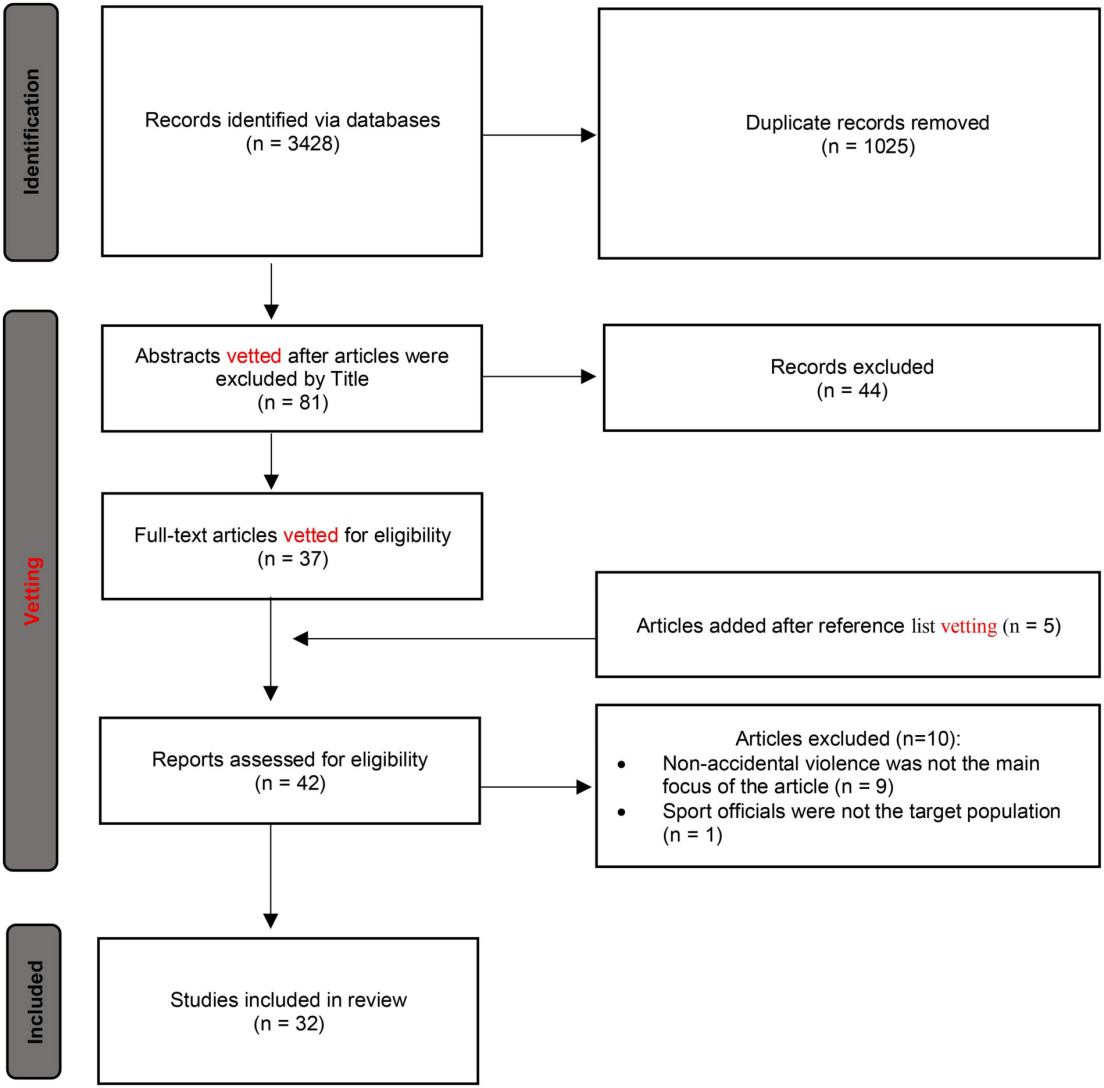
PRISMA flowchart of the search and retrieval strategy of articles.

The search resulted in a total of 3,428 records. After removing the duplicates, 2,403 articles were vetted for eligibility by title and abstract. Firstly, titles and abstracts of articles were vetted independently by two authors through Zotero with the aim of eliminating studies that did not meet the predefined eligibility criteria. Full text reading of articles resulted in a total of 37 articles meeting inclusion, and a further five articles were added upon a bibliographic vetting of identified articles. After the two authors completed an independent vetting of these articles, ten articles were left with a split decision. DH completed a full-text vetting of these articles to settle disagreement between the two reviewers. Following this process, 32 articles remained (see Figure 1). The data were inputted into a data extraction spreadsheet.

### Charting the data

To address the aims of the scoping review, two members of the research team charted the data. The first author created a table to map key findings and to help identify gaps in existing literature. Recommendations from Arksey and O'Malley (23) were used to guide the headings in the table which included: (a) Authors; (b) Publication year; (c) Study location; (d) Population; (e) Study aim/purpose; (f) Methods and methodology; (g) Measurements used; and (h) Key findings. The charted data informed the final stage process which included collating, summarizing, and reporting the data (i.e., the results section herein). After consulting with the second author about the themes in the data, the first author coded the findings and developed the final categories presented in the results inductively.

## Results

Thirty-two articles were included in this review (see Table S1 in Supplementary File for summary of articles and Table 1 below for article characteristics). The following results section synthesizes the included articles to answer the research questions identified earlier in this manuscript. In the section that follows ‘N’ refers to the number of articles while % refers to the percentage of articles. For the purpose of this review, we use the term non-accidental violence when referring to any form of abuse, harassment, bullying, mobbing that is intentional unless a specific type of abuse is reported by authors (e.g., physical, verbal, sexual).

**Table 1:** Study characteristics.

Study characteristics	Number of articles	Percentage of articles (%)
Country of study		
United Kingdom	8	25.0
Spain	4	12.5
Canada	3	9.3
Australia	3	9.3
United States	2	6.2
Turkey	2	6.2
Brazil	2	6.2
Ireland	2	6.2
Sweden	2	6.2
France and Netherlands	2	6.2
Brazil and Spain	1	3.1
United Kingdom and Canada	1	3.1
Sport		
Soccer	19	59.3
Australian Rules Football	2	6.2
Hockey	1	3.1
Baseball	1	3.1
Basketball	1	3.1
Rugby union	1	3.1
Rugby League	1	3.1
Range of sports	6	18.7
Sample size		
<5	1	3.1
5 to 30	13	40.6
101 to 500	6	18.7
501–1,000	4	12.5
1,001+	6	18.7
Did not specify	2	6.2
Sample gender		
Men only articles	3	9.3
Women only articles	5	15.6
Men and women articles	16	50.0
Did not report in article	8	25.0
Methods		
Quantitative	11	34.3
Qualitative	14	43.7
Mixed-method	7	21.8
Publication Year		
Pre-2000	1	3.1
2001–2010	3	9.3
2011–2020	17	53.1
2021+	11	34.3

### The nature of the research

The methods of the 32 studies are synthesized and presented below under the following main headings: (a) Underlying theory; (b) Participant characteristics; (c) Study design and measures; and (d) Study purpose.

#### Underlying theory

Fifteen articles (46%) did not explicitly specify whether an underlying theory or framework informed study aims, the design, or interpretation of findings, while 17 articles did (see Table S2 in Supplemental File for breakdown). Even when theories were not stated, it is often possible to interpret a theory or framework that guided an article (25). In such cases, the first author interpreted the underlying theories or frameworks which guided these articles. A framework was identified if it demonstrated a particular structure consisting of various concepts or variables that were related to a phenomenon, whilst a theory was identified if it provided an explanation of how and why specific variables led to specific events to explain a phenomenon (26).

Eight articles were framed by psychological and/or economic frameworks which included phenomenology, Lazarus and Folkman's transactional theory on stress and coping, cognitive behavioural theory, economic theory of hedonic wellbeing, and principal-agent theory. Through this framework, authors primarily aimed to bring forth the emotional and/or cognitive experiences of sport officials. For example, through phenomenology, Friman et al. (27) revealed how non-accidental violence manifested psychologically (e.g., fear, lack of concentration, lack of motivation), and through cognitive behavioural theory, Kellett and Shilbury (28) connected officials’ perception of non-accidental violence to emotional outcomes.

Nine articles in total were underpinned broadly by a sociocultural framework which included: feminist theory, Foucault's (67) theory of disciplinary power, figurational sociology, and Cohen's Moral panic. These theories framed non-accidental violence toward officials as socially embedded and socially constructed, which arguably went beyond the psychologically-informed articles. That is, those framed by sociocultural frameworks understand that non-accidental violence is not an individual behaviour but rather a cultural phenomenon which reflects gender norms, power dynamics, and societal values. For example, the feminist perspective denoted by Marín-Montín and Bianchi (29) and Forbes et al. (30) highlighted how gendered hierarchies in sport normalized non-accidental violence toward women officials, and Rawlings and Anderson (31) and Gubby and Martin (32) used Foucault's theory of disciplinary power to highlight how women officials internalized gender norms and consequently self-regulated behaviour (e.g., attempted prove themselves in spaces dominated by men).

Nine articles framed non-accidental violence through a psychosocial framework which included: frustration-aggression theory, social information processing theory, intergroup conflict theory, and an ecological systems theory. These theories frame non-accidental violence as socially learned and reinforced within a competitive sporting environment. For example, frustration-aggression used by Dawson et al. (33) and Ackery et al. (34) suggests perceived unfavourable calls from perpetrators of non-accidental violence naturally escalate to aggression. This theory is extended by social information processing theory used by Guérette et al. (35) which suggests that officials then adapt their behaviors (e.g., relaxing calls) to avoid further non-accidental violence. Again, these theories broaden our understanding of non-accidental violence beyond just a psychological construct as they illuminate the role of officials’ social environment in reinforcing non-accidental violence.

Finally, eight articles were framed through an organizational/occupational and/or ecological lens which included: Webb's (8) conceptual model of factors influencing sport officials’ welfare, and a workplace harassment/bullying framework. Through these frameworks it was possible to illuminate the multi-level systems which influence non-accidental violence and support for sport officials. For example, workplace harassment/bullying theories used in four studies heavily problematized non-accidental violence toward sport officials as authors positioned sport as a workplace—a space in which harassment should not exist. An ecological systems theory used by Deal et al. (36) situated officials as part of microsystem (e.g., officiating peers) and a macrosystem (e.g., societal norms, media). Specifically, Marshall et al. (37) used a feminist ecological systems theory which highlighted how gendered systems operating within these levels, influenced the barriers women officials experienced and the role of organizations in legitimizing or mitigating such barriers.

#### Participant characteristics

The participant sample size ranged from four to 4,295. Two studies did not state a participant number as they did not sample sport officials specifically—instead these articles explored non-accidental violence toward officials in a regional and youth amateur soccer league (35), as well as a provincial soccer organization (36). Twenty-seven articles reported the participants’ gender. Although women participants were sampled in 21 articles, their participation was disproportionate compared to men samples—of the 20,267 total participants reported across studies, only 4.1% were women.

Most studies (*N* = 19; 59%) explored non-accidental violence toward soccer referees. Two articles (6%) explored Australian rules football, and the following sports were studied only in one (3%) article each: ice hockey, baseball, basketball, rugby union, and rugby league. Six articles (18%) studied non-accidental violence across a multitude of sports. Participant ages varied considerably, from age 14 to 65+. Ten studies (32%) did not report participant age, and one reported the sample were ‘diverse in age’. Experience and level of sport officials varied between officiating one year to 60 years across articles. Sport officials across articles represented all levels of officiating—from recreational or grassroots to professional. Fourteen articles (43%) did not report experience. Eight articles (25%) did not report the level at which participants officiated. Of those that reported level, the majority sampled a range of levels for their study (*N* = 21; 87%), one (4%) sampled specifically at a provincial level, and two sampled officials at the professional level (8%).

Further participant information was available in some articles. Four studies (12%) included information about sport officials’ ethnicity, religion, or cultural background although some of these statements were broad (e.g., ‘one referee from ethnic minority’). Four studies (12%) included information about current officials’ status (e.g., active or retired)—two of these articles included only active officials and two articles included both active and retired. Two articles (6%) included information about marital status and children, one (3%) article reported sport officials’ income, as well as education background (3%).

#### Study design and measures

Eleven articles employed quantitative methods to establish non-accidental violence prevalence rates or relationships (34.3%). Specifically, nine (28.1%) employed a cross-sectional design, one (3%) used a quasi-experimental and retrospective design, and one (3%) used a retrospective and observational design. The retrospective designs allowed researchers to analyze documents or pre-collected data. Fourteen studies (43.7%) employed qualitative research methods to explore experiences or perceptions of non-accidental violence; 7 of which (21%) employed semi-structured interviews to collect data, one (3%) used a survey, one (3%) focus groups, one (3%) used both semi-structured interviews and focus groups, one (3%) used media stories and focus-group, one (3%) used concept mapping, and two (6%) used ethnographic methods (e.g., combination of observations and interviews). Finally, seven studies used mixed-methods which employed the use of surveys that included open and closed-ended questions.

Thirteen validated questionnaires were used across five quantitative articles and one mixed-method article. These questionnaires aimed to measure distress (e.g., Distress Screener; *N* = 2), anxiety (Generalized Anxiety Disorder Scale; *N* = 2), mental health (Mental Health Continuum-Short Form; *N* = 1) well-being (e.g., Short Warwick-Edinburgh Mental Wellbeing Scale; *N* = 1 and UK Government's Taking Part Survey; *N* = 1), health (Patient Health Questionnaire; *N* = 2), coping (e.g., Coping Styles scale; *N* = 1 and Brief Cope; *N* = 1), optimism (e.g., The Life Orientation Test; *N* = 1), and sexual assault (e.g., The Sexual Experiences Questionnaire; *N* = 1). The majority of these validated questionnaires were developed for general or specific populations, and then adopted and/or adapted by authors for sport officials. In contrast, two articles (6%) used sport official-specific questionnaires: (a) Referees’ Sources of Stress Scale and (b) Mobbing Scale for Football Referees, which respectively explored stress and bullying in sport officials.

#### Study purpose

Eleven articles focused on the identification of prevalence rates and/or frequency of non-accidental violence toward sport officials, fifteen articles explored sport officials’ experiences or perceptions of non-accidental violence, and three studies focused on identifying the types of non-accidental violence experienced by officials; specifically, verbal abuse was the most widely explored (*N* = 19), followed by physical abuse (*N* = 12), sexual abuse (*N* = 4), and three articles focused on identifying the relationships between these types of abuse. Thirteen articles explored the relationship between sample characteristics (e.g., gender, age, experience, level of officiating, marital status, and education status) and non-accidental violence, nine articles focused on the impact of non-accidental violence including its influence on mental health, retention, performance and/or motivation, four articles aimed to understand how officials cope with non-accidental violence, and five articles were interested in perceptions of organizational and/or peer support. Finally, three articles focused on an intervention embedded in soccer organizations, exploring either its effectiveness to curb non-accidental violence or experiences of officiating since the introduction of an intervention.

### Study findings

Findings from the 32 studies are synthesized and presented below under the following main headings: (a) Prevalence rates and types of non-accidental violence; (b) Perpetrators of non-accidental violence; (c) Demographics and risk; (d) Impact of non-accidental violence; (e) Coping; and (f) Organizational support.

#### Prevalence rates and types of Non-accidental violence

Articles which explored prevalence (*N* = 11; 34%) concluded that sport officials had either experienced or witnessed some form of non-accidental violence. Specifically, Gomez et al. (38) reported up to 92.3% of participants experienced some form of non-accidental violence.

Verbal abuse was the most widely reported type of non-accidental violence. Between 63.6% (39) to 94.2% (11) of sport officials experienced verbal abuse, which was characterized by threats of physical violence, intimidation, insulting remarks including racist and sexist comments, mobbing (bullying), yelling, and gestures. Monteiro et al. (40) even noted 16.6% of their sample had been threatened with a firearm in their sample of soccer referees in Brazil. Lishman et al. (10) highlighted that 17.1% of officials experienced verbal abuse via social media. Verbal abuse occurred frequently, specifically, between 28.1% to 49% (41) experienced verbal abuse a few times a season, and between 31.4% (11) to 60% (12) experienced abuse every couple of competitions.

Between 7.5% (10) to 23% (11) of officials experienced physical abuse, characterized by shoving, grabbing, striking, spitting, blocking paths, and hitting. Although physical abuse was less frequently experienced by sport officials directly compared to verbal abuse, 66% of sport officials in Monteiro et al. (40) reported witnessing physical abuse or attempted assaults, and Dawson et al. (33) reported that officials who experienced one form of abuse were likely to experience the other.

Few studies focused on sexual abuse or harassment (*N* = 4; 12%), and these results were not always consistent. This form of abuse was characterized by inappropriate sexual comments about appearance and uniforms, unwanted touching, sexually explicit messages, and sexual coercion. While Gomez et al. (38) reported no gender differences in sexual harassment, Castillo Viera et al. (42) found women scored higher than men when measuring this dimension. Castillo Viera et al. (42) also reported that unwanted sexual attention and coercion occurred rarely in their sample. However, unwanted sexual attention from athletes, men officials, coaches, and spectators, as well as sexual propositioning from men officials to advance career were a salient experience for women officials in Marshall et al. (37) and Rawlings and Anderson (31).

Three articles reported that the rates of non-accidental violence were only increasing. For example, Deal et al. (36) mapped the increase through monitoring disciplinary incidents enacted on sport officials over a five-year period, highlighting a 148% increase in incidents.

#### Perpetrators of non-accidental violence

Athletes, coaches, and spectators were cited as the main perpetrators of non-accidental violence, and often, it was felt that non-accidental violence was a reaction to decisions that athletes, coaches, and spectators disagreed with (27). Non-accidental violence from one of these groups heightened the likelihood of experiencing violence from the other (43, 44). Coaches and managers even instructed athletes to act violently toward officials (40, 43). These works suggest that athletes and coaches hold a perception that non-accidental violence toward officials might influence their decision-making. Guérette (35) supports this, highlighting that baseball teams benefited from resorting to verbal abuse as officials were less likely to make a decision that negatively affect the aggressor's team.

Devís-Devís et al. (45) noted that spectator violence toward officials was mostly sexist or racist. Articles which adopted a gendered lens (*N* = 4; %12) were able to explore this further as they intentionally considered the ways gender influenced non-accidental violence through examining specific sub-cultures within sport and social structures within society. For example, long-held assumptions regarding gender norms led to athlete, coach, and/or spectator perception that women officials were less capable or had less knowledge than men counterparts, despite experience and qualifications (30–32, 37).

Media was also cited as a big influence on perpetrating athlete and spectator non-accidental violence toward officials (40). Media outlets sensationalized non-accidental violence; that is, rather than spotlighting the phenomenon as an issue, it was used to attract larger audiences (29), which was deemed problematic given aggressive actions of elite athletes highlighted on television set examples for spectators and grassroots level athletes (12).

Finally, social agents inside of officials’ sporting organizations were identified as perpetrators of non-accidental violence. Sport officials’ peers exhibited mobbing behaviors due to jealousy and rivalry (46). Further, the organizations which officials operated in also perpetrated non-accidental violence. Firstly, their hierarchal culture or ‘boys club’ facilitated peer-to-peer non-accidental violence (37, 46). The organizations’ lack of adequate sanctions for aggressive behavior from athletes, coaches, and spectators also legitimized the issue, allowing it to persist (8, 13).

#### Demographics and risk

##### Gender

Findings in articles which focused on comparing non-accidental violence between men and women samples were inconsistent. For example, Dawson et al. (33) found that women officials in France faced less non-accidental violence than men counterparts, Rainey (47) found no significant differences in assault rates based on gender, and Gómez et al. (38) reported that men scored higher than women across verbal and physical abuse as well as manipulation, with no significant gender differences found in sexual harassment. It should be noted sample size for women officials in these studies was small compared to men samples.

In contrast, Hacicaferoğlu and Gündoğdu (48) revealed women experienced more mobbing (bullying) behaviors compared to men. It is possible to glean further insights on this through the qualitative articles which had a deeper focus on women officials’ experiences and perceptions of non-accidental violence [e.g., (27, 29, 30, 32)]. These articles highlighted that women officials mostly experienced verbal abuse and sexual abuse, with the verbal abuse women officials received pertaining to gender bias (e.g., sexist comments about their ability to officiate) (27). For example, women officials noted feeling their decisions were consistently undermined and their mistakes judged more harshly compared to men colleagues making them feel like outsiders in their respective sports (30). As such, women officials found themselves navigating the perceived incompatibility of being a woman and perceived as a competent official.

##### Age and experience

Younger officials were more exposed to non-accidental violence (48) due to lack of experience. However, Folkesson et al. (39) reported that older officials with less experience still faced less non-accidental violence than younger officials, suggesting that appearing young and easier to intimidate may be why younger officials are at risk of non-accidental violence (13). In contrast, Gómez et al. (38) identified that their sample of older soccer officials reported higher hostility and manipulation.

In regard to experience, articles present conflicting results. While some reported that officials with more years’ experience encountered more non-accidental violence (33, 38), Webb et al. (12) reported that less experienced officials (between 0 and 5 years) experienced more frequent non-accidental violence. These findings may be explained through inconsistent measurements used across studies. For example, while Brick et al. (11) and Webb et al. (12) explored the relationship between experience and non-accidental violence by asking specifically about frequency (e.g., how often per season/game have you experienced abuse), other articles asked more broadly about total experience with non-accidental violence throughout their careers.

##### Level of competition

Rainey (47) found no significant differences in non-accidental violence based on competition level. Others reported that non-accidental violence most frequently occurred in the lower levels of competition (8, 12). This is explained by factors such as unrealistic expectations, being closer in proximity to spectators, and abusive athlete behavior being modelled by higher levels. Webb et al. (12) explained that at lower levels, behavioral regulation is also more challenging due to less public scrutiny than professional sport and less regulation which allow non-accidental violence toward officials to persist.

#### Impact of Non-accidental violence

The articles which focused on impact (*N* = 9; 28%) concluded that non-accidental violence had negative consequences for sport officials. The only exception was Kellett and Shilbury (28) who reported that non-accidental violence was not particularly aversive in their sample of professional and semi-professional Australian Rules football players.

##### Mental health impact

Mental health effects such as depression and anxiety were reported by up to 50% of participants (31). Although physical abuse was less frequent than verbal abuse, it led to higher distress rates when it did occur (11). Some anxiety arose from fear of being assaulted by athletes, coaches, or spectators due to a lack of security (40). Low self-esteem (27) and imposter syndrome (31) occurred due to non-accidental violence, while women officials also reported lower well-being than men (49).

##### Retention

Although Kellett and Shilbury (28) suggested there was no evidence that non-accidental violence led to attrition, all other articles reported that non-accidental violence led officials to quit or consider quitting. Specifically, 7.7% of participants cited non-accidental violence as the main reason for considering quitting (11). In response to the consistent verbal abuse, participants felt the complexity of officiating was undervalued and that their roles were not fully appreciated, also contributing to attrition (13). Dawson et al. (33) suggested years of experience officiating mediated retention, and that those with more years in the physically and mentally demanding profession were more likely to quit due to the toll that the role had taken overtime.

While some officials experienced a decline in mental health and pondered quitting due to abuse, many ultimately decided to remain in their roles. Cited motives to stay included passion for the game (27, 40) and opportunities for socializing and meeting people (27, 28). This suggests that the positives associated with officiating coupled with high resilience (49) offset the non-accidental violence some officials receive.

#### Coping

Officials employed various problem- and emotion-focused coping strategies in response to non-accidental violence. Some problem-focused strategies included penalties and send-off calls (45) and communicating decisions effectively with athletes and coaches (13, 27); the latter being reported as a successful strategy to reduce aggression (27). Monteiro et al. (40) also reported that officials often relaxed the rules at an amateur level to ensure good competition conduct and protect themselves from non-accidental violence.

Emotion-focused strategies included smiling, not taking verbal abuse personally, and managing fear (45). Avoidance-coping included minimizing or tolerating non-accidental violence. Those who minimized the non-accidental violence received viewed it as a normal part of the job accepting that it was inherent to their role (28, 50). Participants in Radziszewski et al. (13) noted non-accidental violence was acceptable up to a certain point (e.g., insulting the uniform was accepted but not personal attacks). However, Bressan et al. (43) warned that the acceptance of non-accidental violence as “part of the job” risks developing a sporting culture which legitimizes non-accidental violence and allows it to go unreported. A further avoidant-coping strategy included ignoring behaviors such as abusive comments, although this strategy sometimes escalated the non-accidental violence (13). Women officials often self-regulated their behavior (e.g., apologized for decisions) to avoid non-accidental violence (31). Although avoidant-coping strategies were commonly reported to manage non-accidental violence, these were also associated with poorer mental health outcomes such as anxiety and depression (10).

Finally, officials found non-accidental violence from athletes and coaches more difficult to cope with than from spectators, as athletes and coaches were seen as more knowledgeable compared to an anonymous and lesser knowledgeable group (e.g., spectators) (39), putting officials’ ability into question.

#### Organizational support

Articles presented conflicting results regarding the level of support received from their sporting organization in response to non-accidental violence. Many officials felt supported by their organizations, receiving supportive follow-up phone calls after an incident (13), actions from organizations which reinforced zero-tolerance policies for offenders such as suspensions or fines (51), or in rare cases, legal charges and convictions (47).

In contrast, some sport officials perceived they were solely responsible for managing non-accidental violence, as their complaints to their respective organizations were ignored leading to perceptions of insufficient support (43). For example, sport officials reported decisions were altered and bans were reduced leading to feelings that their organizations sided with perpetrators (8). Officials also experienced repercussions for reporting non-accidental violence or supporting fellow officials, a lack of help with complex incident reports, and inadequate sanctions against perpetrators (27, 52). Specifically regarding the latter, Rainey (47) reported that 64% of dangerous assaults (e.g., choking or hitting with bats) had consequences for perpetrators while only 11% of minor assaults (e.g., pushing or grabbing) had serious consequences, further supporting perceptions of insufficient support.

Some organizations had formal processes of reporting and support networks in place; however, these were not always sought by officials due to concerns about their adequacy (50), particularly from young or inexperienced referees (8, 52). All intervention studies highlighted that the programs’ respective impact was diminished by inconsistent application across different regions of a country, and/or lack of punitive measures for perpetrators (12, 51, 52). While organizations were said to have the tools to enforce behavior standards to curb official non-accidental violence, this was not always undertaken (51). As such, sport officials expressed doubts that non-accidental violence could be completely eradicated as it was ingrained in sport culture (12). Ultimately, poor organizational support in response to non-accidental violence led officials to experience feelings of isolation, self-doubt, abandonment, unfulfillment, and some pondered quitting (13, 33, 46).

Some organizations provided training to help support officials. However, some officials found it unfair that they were required to attend mandatory training to maintain their roles while perpetrators including athletes, coaches, and spectators were not held to the same standard (12). Therefore, education for perpetrators was identified by up to 66% of sport officials as a key contributor to changing abusive behaviors from perpetrators toward sport officials (12, 40). Officials noted training specifically on conflict management, stress management, and decision-making would also help support them (8). Other officials suggested the infrastructure for officiating was inadequate, which facilitated non-accidental violence toward officials (40). For example, courts or stadiums lacked proper barriers—which meant spectators were close to officials (40), and changing rooms were masculine spaces exclusive of women officials making them feel unsafe and exposed to some abusive men officials (31).

## Discussion

To the best of our knowledge, this is the first review to explore and synthesize research specifically focused on non-accidental violence toward sport officials across types of officials and sports. This scoping review provided an overview of the nature of non-accidental violence in sport official research as well as insight into types of non-accidental violence, perpetrators of non-accidental violence, demographics and risk, the impact of non-accidental violence, coping, and organizational support. The results in this review highlight that non-accidental violence is a pervasive concern with up to 94.2% of samples experiencing some form of non-accidental violence. Its impact is wide-reaching, affecting the psychosocial wellbeing of sport officials across sports, ages, levels, and gender. The results of this study, as well as findings from other sport officiating and athlete literature suggest non-accidental violence is rooted in a win at all costs ideology (62). This ideology coupled with limited coping and poor support can have alarming, long-lasting consequences for some officials as they can experience depression, anxiety, and low self-esteem. These consequences should also concern sporting organizations, as they risk a continued decline in retention if the problem is not addressed. In the rest of this section, we critically discuss our results, identify the gaps in the research, and in doing so, provide recommendations for future researchers to prioritize, to ensure a robust body of literature.

### Prioritizing vulnerable and marginalized groups

The articles’ findings offer a window into the characteristics of sport officials and the relationship with risk for non-accidental violence. For example, authors identified younger officials were at risk of non-accidental violence. Some perpetrators specifically targeted young officials due to a perception they are easier to intimidate and will thus change decision in their favor, which aligns with research reported about coaches enacting non-accidental violence toward young athletes (4, 68). Echoing Brackenridge and Kirby (69), non-accidental violence toward young officials is allowed to occur within sport in part because minors (i.e., under-18s) in sport are not treated as minors first, but rather viewed by their role first (e.g., athlete, official) meaning the same precautions are not inherently held for these under-18s that are typical in a ‘normal’ workplace. From an occupational lens, this is highly problematic, not least from a safeguarding and welfare perspective. Several initiatives in sport now exist which are seeking to dismantle abusive practices directed toward young sport officials. For example, the green armband initiative was adopted in Canada in hockey, and England in soccer as a support mechanism to protect under 18-year-old officials from player, coach, and spectator non-accidental violence. In the program, any official under the age of 18 wears one green armband to identify them as a child. Although some studies have explored the effectiveness of interventions to target non-accidental violence in professional leagues, as seen in this review [e.g., (12, 51)], one evaluating a program specifically for young officials from their perspective has yet to be conducted.

There were limited studies that mentioned ethnicity or sexual orientation, nor explored how such intersectional identities influenced non-accidental violence experiences. Those from marginalized groups bear a greater risk for experiencing non-accidental violence (53). As such, research exploring the experience of ethnic (e.g., Indigenous or Black) or sexual orientation (e.g., LGBTQ+) minority officials will be useful in shedding light on intersecting identities and non-accidental violence, and the role of social and organizational structures within sport in maintaining and/or deterring non-accidental violence. The inclusion of minority groups will be facilitated through the adoption of sound epidemiological methods such as robust demographic questionnaires to better understand the characteristics of officials. In doing so, practitioners can create specific interventions tailored toward minority officiating groups.

There were also research gaps in regard to gender, as women represented only 4.1% of participants. This contrasts with findings on women athletes and non-accidental violence, and perhaps represents the gender trends seen in general sport officiating literature (24) which has not prioritized the recruitment of women samples. When women samples were used, these were often set against men samples with narrowed research questions and results which lack context about the role of wider sporting structures in influencing women's experiences of non-accidental violence. Articles that focused on the relationship between gender and non-accidental violence highlighted how sociocultural gendered norms led to misogynistic sport cultures where women officials were consistently treated as less than men counterparts. Future studies should extend this promising work through focused grants which explore further how gendered norms and women official-specific barriers may influence the experience of non-accidental violence [e.g., sexualization of women bodies through (social) media portrayal, tokenism, underrepresentation in men dominated sports]. Finally, although we understand that certain groups might be more vulnerable to non-accidental violence, we still know little about the antecedents of non-accidental violence, which we therefore recommend as an area of inquiry for future researchers.

### Diversifying research questions and methodological choices

In terms of the research questions asked by researchers regarding impact, the links between non-accidental violence and retention were well-established by a small subset of studies (33, 49). However, a greater examination into the impact of non-accidental violence on officials’ mental health is needed. Sport officials experience a series of mental health concerns [see Carter et al. (9) for review]; however, more insight is needed into how (and if) non-accidental violence contributes to poor mental health. For example, post-traumatic stress disorder (54), self-harm (55), disordered eating, and body image concerns (56) have been reported in the athlete non-accidental violence research. In line with recommendations by Cater et al. (9), a thorough investigation into different mental health concerns is missing in the current body of literature on non-accidental violence toward sport officials.

Thirty-seven percent of articles used quantitative research designs to establish prevalence, magnitude and risk of non-accidental violence. While these articles heighten our understanding of the severity of non-accidental violence, there is room to broaden the scope of research targeting this phenomenon through the adoption of more diverse methodological choices. Again, this can be done through focused research that seeks to understand non-accidental violence from an epidemiological perspective exploring how demographic characteristics concerning race, ethnicity, sexual orientation, class, and (dis)ability interact with non-accidental violence.

Further, although qualitative methods were adopted by the majority of articles (43%), the methodological choices at times restricted participant voices. For example, some articles presented limited rich data due to lack of participant quotes as these articles chose to adopt restricted methods (e.g., narrow research questions, surveys) which were unable to delve into participant experiences. Such methods exemplify ‘small q’ qualitative research (57) which refers to the categorization of participant experiences into frequencies or descriptive themes lacking context. In contrast, there were mixed-method and qualitative studies included in this review which adopted richer methods. From these articles it was possible to understand how some organizational and social influences impacted non-accidental violence (e.g., power dynamics, lack of support structures). Therefore, its use can continue to advance insight further into the psychosocial and cultural influences which influence non-accidental violence; that is, how the wider sporting culture facilitates non-accidental violence toward officials.

By no means is the adoption of ‘small q’ methods inherently bad. However, it is important to consider the complex ethical challenges that arise with researching a sensitive topic such as non-accidental violence (58). Given the likelihood of distress to participants, the topic warrants careful selection of data collection and analysis choices which consider such sensitivities—a practice vital for researching in a trauma-informed manner (59). For example, researchers may choose to adopt rich data collection methods such as loosely structured interviews or creative methods such as photo elicitation or expressive writing that allow the participant autonomy in disclosing their experiences. Further, rich data analysis methods such as narrative, reflexive thematic analysis, or creative non-fiction are appropriate to study non-accidental violence as they highlight participant accounts in a manner which represent their lived experiences.

### Considering an organizational Lens

Finally, review findings highlighted that by simply existing in a sporting environment, sport officials were at-risk for experiencing non-accidental violence. This suggests there are inadequate safeguarding practices for organizations’ employees. Much like with any occupation, some sporting organizations (e.g., in Canadian provinces and territories) that hire officials have a legal responsibility to protect officials working within the space. All sports organizations should have policy in place to protect their employees which include actionable practices such as punishments for perpetrators, formal support, confidential reporting pathways, and policy. Future researchers could adopt multi-level designs which consider a systems approach to non-accidental violence, framing it as an organizational concern (e.g., how power structures and different people within a sport system contribute non-accidental violence) rather than framing it as an individual problem, which articles within this review have already begun to shine light on [e.g., (12)]. Although only few articles explored non-accidental violence using a sociocultural and/or organizational lens, these articles highlighted that the use of sociocultural and/or organizational theoretical frameworks privilege experiential insights so careful consideration of theory is needed to appropriately engage in this work.

### Limitations

Although the research presents a thorough synthesis into non-accidental violence in sport officials, it is important to acknowledge study limitations. Firstly, studies were not included unless non-accidental violence (abuse and harassment) was stated in the article's aims, meaning articles where non-accidental violence was discussed in the results but not as a specific aim were excluded. Although this risked losing potentially relevant insights, the decision was made as the articles which included non-accidental violence in their aims were likely to use frameworks, methods of data collection and analysis which explicitly capture in-depth insight into the phenomenon. In doing so, non-accidental violence is at the forefront, rather than described as a secondary observation which may have weakened comparability across studies. Secondly, although we acknowledge officials experience different demands and experiences based on gender, sport, and level, which at times made synthesis difficult, a decision was made to include all types of officials in this review to gain a better understanding of non-accidental violence in the population as a whole and to highlight the gaps in officiating characteristics, thus ensuring a full-spectrum review. Third, the included articles were largely concentrated in Europe, North America, and Australia and interactor sports which might limit the opportunities for generalizability across regions and different sports. Finally, a strength of this review was the inclusion of articles other than solely English-language articles. However, specific databases or search terms in those languages were not used. Despite this, three Spanish or Portuguese articles were identified, and their reference lists were scanned to ensure as many relevant articles as possible were captured.

## Conclusion

A review of literature was needed to understand the scope of the academic coverage concerning non-accidental violence toward officials across sports. We found 32 studies which provided insight into the prevalence and risk of non-accidental violence toward sport officials, the perpetrators of non-accidental violence, its impact, and how officials cope with non-accidental violence. Allied to this, through the synthesis of articles’ methods, we were able to identify gaps in the current body of literature. Echoing the recent expert statement by Webb et al. (60) we argue that there is a need to focus on vulnerable or marginalized officiating groups such as underage officials, women and those from ethnic minorities. Future researchers must carefully consider theoretical and methodological decisions. By highlighting these gaps, we provide potential future avenues for researchers to explore in order to advance work within the field.
