# Supplementary material for: Non-accidental violence toward sport officials: a scoping review
Source: Front Sports Act Living. 2026 Mar 25;8:1765769. doi: 10.3389/fspor.2026.1765769 (PMC13057381; doi:10.3389/fspor.2026.1765769)
Supplement: Supplementary file 1 [file Table1.docx]

Appendix

[Table 1. Search terms 2](#_Toc197522448)

[Table 2. Article and participant characteristics 3](#_Toc197522449)

# **Table 1.** Search terms used in systematic search.

| Database Searched | Number of Articles | Search Terms | Search Fields |
| --- | --- | --- | --- |
| PsycINFO | 341 | “abuse” or "physical" or "psychological" or "sexual" or "neglect" or “harassment” or “non-accidental violence” or "violence" or “maltreatment” or “bully*”  AND “sport*”  AND “officia*” or “referee*” or “judg*” or “umpir*” | Abstract  All Text  All Text |
| SPORTDiscus | 694 | “abuse” or "physical" or "psychological" or "sexual" or "neglect" or “harassment” or “non-accidental violence” or "violence" or “maltreatment” or “bully*”  AND “sport*”  AND “officia*” or “referee*” or “judg*” or “umpir*” | Abstract  All Text  All Text |
| Web of Science | 688 | “abuse” or "physical" or "psychological" or "sexual" or "neglect" or “harassment” or “non-accidental violence” or "violence" or “maltreatment” or “bully*”  AND “sport*”  AND “officia*” or “referee*” or “judg*” or “umpir*” | Keywords Plus  All Fields  Topic |
| PubMed | 1,705 | “abuse” or "physical" or "psychological" or "sexual" or "neglect" or “harassment” or “non-accidental violence” or "violence" or “maltreatment” or “bully*”  AND “sport*”  AND “officia*” or “referee*” or “judg*” or “umpir*” | MeSH Subheading  All Fields  All Fields |
| Total | 3,428 |  |  |
| Total after duplicates removed | 2,403 |  |  |

# **Table 2.** Article and participant characteristics

| Author | Study purpose | Study location | Participant information | Methods | Underlying theory | Key Findings |
| --- | --- | --- | --- | --- | --- | --- |
| Ackery et al. (2012) | To examine the perceptions and roles of referees about violence and injury in hockey games. | Canada | N=632 (M=613; F=19); Sport: Hockey  Age: 36 years (mean) Experience: >20.1 years (25.9%) 10.1-20 years (34.0%) 5.1-10 years (27.1%) 5 years(13.9%) | Quantitative; Cross Sectional; Surveys | No theory explicitly stated. Psychosocial framework, specifically, frustration-aggression identified. | 90% of referees reported that they were targets of aggression and anger. 55% had been involved in hockey games where aggressive behavior resulted in the referee losing control of the game. |
| Bressan et al. (2019) | To identify the violence perceived by referees in youth school games. | Brazil | N=19 (M=15; F=4); Sport: Hand ball, basketball, futsal and volleyball Age: 32 (mean) Experience: 7.5 (mean) | Qualitative; Semi-structured interviews;  Analysis method not specified (content analysis described) | No theory explicitly stated. Sociocultural framework identified. | All referees reported experiencing or witnessing some form of violence (verbal, physical, psychological). Many referees normalized the violence they faced. Verbal abuse (including racist and sexist comments) was the most common, followed by physical. Athletes, coaches and fans were perpetrators of violence, with some coaches instructing players to act violently toward referees. Female referees reported sexist treatment and gender discrimination. Organizational support was minimal, and punishments for offenders was limited. |
| Brick et al. (2022) | To investigate the prevalence and frequency of verbal and physical abuse and explore the relationships between abuse, distress, mental health outcomes, and intentions to quit. | Ireland | N=438 (M=434; F=4); Sport: Gaelic football, Hurling, Ladies Gaelic football, Camogie, Rounders, Handball  Age: 45.23 ± 11.11 (mean and SD) Experience: 11.68 ± 9.23 (mean and SD) Current highest level: Club-level adult (n = 260; 59.36%); Intercounty-level adult (n = 90; 20.55%); Intercounty-level underage (n =53; 12.10%);  Club-level underage (n = 35; 7.99%). | Quantitative; Cross-sectional; Surveys | Organizational/ Environmental framework, specifically, Webb et al.’s (2020) conceptual model of factors that influence sports officials’ welfare. | 94.29% of Gaelic Games match officials experienced verbal abuse during their careers and 23.06% of officials reported physical abuse. 43.3% experienced verbal abuse a couple of times a season and 31.8% experienced abuse every couple of games. Physical Abuse was less frequent but lead to higher distress when it occurred. 48.63% of officials considered quitting due to abuse, and 14.16% were thinking of quitting. 7.76% cited abuse as the main reason for considering quitting |
| Castillo Viera et al. (2019) | To analyze the sexual harassment behaviors in football refereeing. | Spain | N=1921 (M=1783; F=138) Sport: Soccer | Quantitative; Cross-sectional;  Surveys | No theory explicitly stated.  Organizational/ Environmental framework, specifically, workplace harassment theory identified. | Sexist hostility was the most frequently experienced. Sexual hostility occurred with some frequency. Unwanted sexual attention and sexual coercion occurred rarely. There were gender differences in all types of sexual harassment, with women scoring higher in all four compared to men. |
| Cleland et al. (2018) | To examine referees experiences of officiating since the implementation of a Respect programme in 2008 by the English Football Association. | UK | N=2056 (M=1984; F=72); Sport: Soccer Age: <17 (18.4%), 18-21 (8.2%), 22-25 (4.1%), 26-35 (10.1%), 36-45 (11.8%), 46-55 (23.4%), 56+ (24%).  Level: Level 7 (junior) (33%), Level 5 (29.8%), Level 6 (13.5%), Level 1 (national list)(0.4%) and English Premier League (0.3%).  Experience: 22.7% of the sample had refereed for <2 years (22.7%), 3-5 years (19.6%), 6-10 years (17.1%), 11-15 years, (11%), 16-20 years (9.1%) and 21+ years (20.5%). | Mixed method,  Surveys (closed and open-ended questions) | No theory explicitly stated.  Organizational/ Environmental framework identified. | Despite the Respect programme, the majority of referees face verbal abuse regularly, and 20% encounter physical abuse. The programme was not consistently enforced across the 51 County FAs as some areas applied stricter and more effective measures than others. Referees called for more consistent and stronger punishments for abuse. The level of support from County FAs varied with some providing excellent support, while others did not. |
| Cleland et al. (2015) | To investigate the effectiveness of the Respect Campaign by examining its impact on a sample of referees from one English County FA in the Midlands. | UK | N=11 (M=10; F=1); Sport: Soccer Officiating at level 1 to level 7  1 referee from an ethic minority | Qualitative Semi-structured interviews;  Thematic analysis | No theory explicitly stated.  Organizational/ Environmental framework identified. | The campaign's impact was weakened by inconsistent application and lack of stronger punishments (e.g., fines, suspensions). Participants report the FA and County FAs need to better utilize their existing tools and offer more support to referees, including effective training for new referees |
| Dawson et al. (2022) | To investigate the factors that are associated with the verbal and physical abuse of the referees and also the association of this abuse with the intentions of referees to quit officiating. | France and Netherlands | N=4295 (M=4177; F=118); Sport: Soccer Officiating at all levels (i.e., grassroots to top domestic divisions) | Quantitative;  Cross-sectional; Surveys | Economic framework, specifically, Principal and Agent theoretical perspective, and psychological theory, specifically,  Frustration-aggression theory | Referees who experience one form of abuse (verbal or physical) are likely to face the other. Referees with more years of experience encounter more abuse. Female referees face less abuse than men in France. There was a significant correlation between years of experience and a higher probability of referees intending to quit. Both forms of abuse increased intentions to quit in both countries. |
| Deal et al. (2018) | To document the frequency and types of disciplinary incidents directed toward men’s soccer referees | Canada | A provincial soccer organizations | Mixed method (although only the quantitative extracted); Retrospective and observational Document analysis | Psychosocial framework, specifically, ecological systems theory (Bronfenbrenner, 2005; Bronfenbrenner & Morris, 1998) | Over a five-year period, ~55,000 soccer games were played and a total of 98 disciplinary incidents were reported across these games. From 2010 to 2015, there was an increase in disciplinary incidents. 56.12% of incidents occurred in the lower levels. The most frequently reported offences were: foul or abusive remarks, threatening an official, physical abuse (e.g., striking or spitting, shoving or grabbing an official). |
| DevÌs-DevÌs et al. (2021) | To know the types of aggression and violence referees experience, and the strategies they use to manage these behaviors. To understand the personal and contextual characteristics involved in the aggression, violence, and coping experienced by Spanish football referees. | Spain | N=8 (M=4; F=4); Sport: Soccer Age: 19-34 years of age Experience: 2-17 years of experience.  Level: Grassroots youth football in both men's and women's football.  Spanish (N=6); Moroccan (N=2) | Qualitative Semi-structured interviews;  Thematic analysis | No theory explicitly stated.  Transactional perspective on stress and coping (Lazarus & Folkman, 1984) identified. | Most of the aggressions from spectators were of a sexist nature, as well as some racist aggressions. Problem and emotional-focused coping strategies were identified which included penalties and send-off calls to players and coaches. Smiling and not considering insults as a personal matter were two emotional-focused coping strategies used toward spectator aggressions. |
| Downward et al. (2024) | To develop understanding of the relationships between physical and nonphysical abuse, the intention to quit of referees and their well-being | UK and Canada | N=895 (M=823; F=72); Sport: Soccer; Age: 45 (mean) Experience: 3-27 years Level: Level 3 + (5%) Income: £2,142 per month (mean). 82% received remuneration for their activity; Partner and/or children (76%); Education: Higher education or equivalent (65%) Active officials: 43% | Quantitative;  Cross-sectional; Surveys | Psychology/economy framework, specifically, economic theory of hedonic wellbeing. | Both physical and nonphysical abuse were associated with referees' poor well-being and increased intentions to quit. Nonphysical abuse was more common. While some referees experience a decline in well-being due to abuse, many chose to remain in their roles, suggesting a level of camaraderie with colleagues. Male referees report higher well-being and lower quitting intentions than female referees, suggesting greater abuse and lesser support faced by women officials. |
| Folkesson et al. (2002) | To find out whether and to what extent soccer referees are exposed to threat and aggression from soccer players, coaches/trainers, and the watching public, and to examine how threat and aggression influence referees concentration, motivation, and performance. | Sweden | N=107 (M=107; F=0); Sport: Soccer Age: 37 + 12.87 (mean and SD) Level: District level (85%), Soccer Association level (15%), Division 6 (57%), Division 4 (14%), Division 5 (14%), Division 3 (22%). | Mixed-method;  Cross-sectional; Surveys | No theory explicitly stated.  Psychological framework, specifically framed by the dispositional optimism and coping model. | 72.9% of officials reported experiencing some form of threat or aggression, with verbal aggression being the most common (63.6%). Physical aggression was less frequent (15%) though 35.1% had been threatened with physical violence. Older referees with less experience faced less aggression than younger referees. Referees found aggression from players and coaches more difficult to cope with than aggression from spectators. |
| Forbes et al. (2014) | To explore the experiences of female (association) football officials who officiate in amateur men's and boy's football matches | UK | N=4 (M=0; F=4); Sport: Soccer Age: 16-33 years Level: Level 4 (County-level and semi-professional assistant referee) to level 7 (Local amateur-level referee); Experience: 2-7 years Ethnicity: All white British and middle class | Qualitative; Ethnographic (Participant observations, informal conversations, semi-structured interviews) | Sociocultural framework, specifically, Feminist theory. | Stereotypes assumed female officials were incompetent, regardless of qualifications. They felt they were representing all women, which added pressure to perform perfectly to challenge stereotypes. Their mistakes were judged more harshly than males, and their correct decisions were undermined. Many referees downplayed abuse, excusing it as part of football culture. Abuse had emotional consequences and some thought about quitting. |
| Friman et al. (2004) | To investigate the threats and aggression directed at soccer referees, and to examine the reasons why people choose to continue as soccer referees. | Sweden | N=7 (M=4; F=3); Sport: Soccer Age: 18-53 years Experience: 15.4 ± 3.9  Level: The majority have refereed at both a district and football association level.  All active officials | Qualitative; Semi-structured interviews;  Descriptive phenomenology | Psychological framework, specifically, phenomenology. | Referees identified players, coaches, and spectators as the main sources of verbal aggression. Female referees experienced verbal aggression due to gender bias. Some referees expressed feelings of insecurity, depression, and a desire to quit. Strategies to cope with this included staying calm, not taking threats personally, and communicating decisions effectively. Despite the challenges, referees continued due to their passion for the game, opportunities for travel, and meeting people. |
| Fuente et al. (2019) | To examine how the types of aggression shown by three groups towards the referee are related to one another, and how they are perceived by the referee, in amateur football | Spain | Regional and youth amateur soccer league | Mixed-method;  Cross-sectional; Surveys | No theory explicitly stated. | Players, coaches and spectators act independently of one another. Despite this, referees believed that if they experience aggressive behaviors from one of these 3 groups, they are more likely to experience aggression from the others as well. |
| Gomez et al. (2019) | To analyze whether workplace bullying behavior occurs in football refereeing, to observe whether age is a determining factor in covert hostility, manipulation, and physical hostility, and to determine whether years of experience influence the appearance of these behaviors. | Spain | N=183 (M=89; F=94); Sport: Soccer Age: 23.30 ± 4.85; Level: Grassroots Football (3.3%), New Entries (19.7%), Official Football (26.8%), Provincial Football (29%), Honor Division (10.4%), 3rd Division Assistant (1.6%), 3rd Division (6.6%), either 2nd Division B, 2nd Division, or 1st Division Assistant (0.5%), 1st Division (1.1%). | Quantitative;  Cross-sectional; Surveys | No theory explicitly stated.  Organizational/ Environmental framework, specifically, workplace harassment lens identified. | 92.3% of officials experienced some form of harassment. Men scored higher than women in verbal hostility, covert hostility, manipulation, and physical hostility. No gender differences were found in sexual harassment. Older referees reported higher harassment levels. More experience in a division meant more exposure to bullying. |
| Gubby & Martin (2024) | To investigates the treatment of female officials by players, supporters and team managers, and discusses how female officials consider the way they present themselves and continue to work in this space. | UK | N=9 (M=0; F=9); Sport: Soccer Level: From grassroots level to semi-professional men's league | Qualitative; Ethnographic (Participant observation, Interviews, Questionnaires); Thematic Analysis | Sociocultural framework, specifically, Foucault’s (1979) theory of disciplinary power | Experiences of sexist language and abuse were related to traditional gender roles. Women expressed the pressure of representing all women football officials. Despite these obstacles and the frequent 'othering' of women referees within the football environment, their persistent involvement and challenges to actions by enactors, demonstrated their resistance to dominant gendered discourses. |
| Guérette et al. (2024) | To examine the influence of excessive criticism on MLB umpires decisions related to the strike zone. | USA | N=110; Sport: Baseball | Quantitative; Quasi-experimental (Retrospective observational study); Precollected data | Psychosocial framework, specifically, social-information processing (Salancik & Pfeffer, 1978) | After an instance of criticism, umpires were less likely to call strikes against batters from the aggressors team, and were more likely to call strikes against batters from the opposing team. Player Characteristics (e.g., all star status, performance level, years experience, all star appearances) did not influence how umpires responded after criticism. |
| Hacicaferoğlu & Gündogdu, (2014) | To determine the level of psychological mobbing behaviors that the football referees within Turkey Football Federation are exposed to. | Turkey | N=374 (M=344; F=29); Sport: Soccer; Age: 38-42 age (4.5%); 33-37 age (35%); 28-32 age (36.6%); 23-27 age (22.2%); 18-22 age (1.6%) Level: a variety of leagues Marital status: 41.4% single; 58.6% married | Quantitative;  Cross-sectional; Surveys | No theory explicitly stated.  Organizational/ Environmental framework, specifically, workplace harassment lens identified. | Referees were exposed to various mobbing (bullying) behaviors at lower to medium levels (although not severe). The following experienced more mobbing behaviors: women compared to men, married referees compared to single referees, the referees with postgraduate education compared to the other educational groups, the referees of 23-27 age group compared to the other age groups, the referees of 1 to 4 tenure compared to other tenure groups. |
| Kellett & Shilbury (2007) | To explore long-term umpires' experiences of umpiring in Australian Rules football. | Australia | N=22; Sport : Australian Rules Football  Age: 17-65+ Experience: 3-35 | Qualitative; Semi-structured interviews; Analysis not specified (thematic analysis described) | Psychological framework, specifically cognitive behavioural theory | Umpires routinely reframe abuse, considering it to be a normal part of their role. Abuse was not deemed to be aversive, and there was no evidence that it contributed to attrition. Umpires enjoyed the social world they share with other umpires, and identified social interactions among umpires as a key reason for continuing. |
| Lishman et al. (2024) | To explore the prevalence and frequency of abuse experiences over a single season, to determine the impact of abuse and other stressors on sports officials' mental health, and to understand the mental health impact of strategies utilised by sports officials to cope with abuse experiences. | Ireland | N=303 (M=301; F=2); Sport: Gaelic football, Ladies Gaelic football, Camogie, Handball Age: 45.19 ± 12.28 Experience: 11.37 ± 9.67 Highest Level: Club-level adult (63.04%); Club-level underage (11.88%); National Support Panel (10.56%); National Panel (10.23%); Provisional Panel (4.29%) | Quantitative;  Cross-sectional; Surveys | Psychological framework, specifically, transactional perspective on stress and coping (Lazarus & Folkman, 1984) | 88.11% of officials reported experiences of verbal abuse, 7.59% physical abuse, and 17.16% social media abuse during the previous season. Greater use of avoidance-cognitive and approach-oriented coping was associated with higher distress and poorer mental health outcomes following verbal abuse. For social media abuse: self-blame, planning, and behavioural disengagement were associated with poorer mental health. |
| MarÌn-MontÌn et al. (2024) | To analyse the media coverage of violence against female referees in Brazil and Spain | Brazil and Spain | N=7 (M=0; F=7); Sport: Soccer | Qualitative; Media analysis and focus groups; Critical discourse analysis | No theory explicitly stated.  Sociocultural framework, specifically, feminist theory identified. | Media primarily focuses on the most visible and sensationalized forms of aggression, neglecting the lack of recognition for female referees' work. Spanish news repeatedly highlights verbal abuse, spotlighting aggressors rather than addressing the root problem. Physical violence is more prominent in Brazilian coverage. Participants experienced mental health effects such as fear and anxiety. |
| Marshall et al. (2023) | To examine the most important and prevalent barriers that women referees and officials in Australian basketball face. | Australia | N=29 (M=0; F=29); Sport: Basketball | Qualitative; Concept Mapping | Psychosocial/ ecological framework, specifically, socio-ecological framework (based on Ecological systems theory) with a feminist lens (Burton & Lavoi, 2016) | Barriers related to sexism, lack of diversity, and systemic exclusion (e.g., the "boys club") were rated as the most frequently experienced. |
| Monteiro et al. (2014) | To identify and analyse different kinds of violence suffered by a group of amateur referees in Juiz de Fora (Brazil), their subjective perceptiveness on these processes, as well as the influence on their performance and professional practice. | Brazil | N=12 (M=12; F=0); Sport: Soccer | Qualitative; Semi-structured interviews; Discourse Analysis | No theory explicitly stated. Sociocultural conceptual framework identified. | 66.6% of referees reported witnessing physical violence or attempted assaults from players. 50% mentioned experiencing violence or threats during matches, and 41.6% felt unsafe as security was poor. 50% reported verbal abuse from fans caused anxiety. The infrastructure for refereeing was inadequate (e.g., courts lacking proper barriers and changing rooms not meeting standards). 50% of participants believe that more experience led to better decision-making. 83.3% reported the media had an influence on player and fan behavior. |
| Polat et al. (2017) | To determine the psychological violence and pressure faced by football referees in Turkey and to disclose the reasons of the events. | Turkey | N=17 (M=17; F=0); Sport: Soccer Age: 28-36 Experience: 10-15 years Level: Different levels in Turkish Football Federation. National (57%), Regional (41%), Provincial (29%) | Qualitative Phenomenology;  Focus groups;  Descriptive qualitative analysis | No theory explicitly stated. Organizational/ Environmental framework, specifically, workplace harassment lens identified. | Referees face consistent psychological stress both from within their organizations and from external stakeholders, especially for younger referees which can result in performance anxiety, demotivation, and quitting. Psychological violence came from both higher-ups and peers as well as outside the referee organization including: Fans, players, coaches, media, and club managers. |
| Radziszewski et al. (2023) | To document the experiences of abuse in young officials (14-20 years). | Canada | N=27 (M=21; F=6); Sport: Soccer, Hockey, Volleyball, Baseball, Basketball, Softball Age: 14-15 (18.5%), 16-17 (66.7%), 18-20 (14.8%)  Experience: 1 year (14.8%), 2 years (25.9%), 3 years (25.9%), 4 years (22.2%), 5+ (11.1%) | Qualitative; Semi structured interviews; Thematic analysis | Psychosocial framework, specifically, intergroup conflict theory | Physical abuse was rare. Many officials felt supported by their clubs or associations; they received follow-up calls or emails reinforcing zero-tolerance policies. However, some felt isolated or abandoned, noting a lack of help with complex incident reports or feeling that their association sided with the abuser in some cases. Officials used problem-focused strategies (explaining decisions or issuing warnings), emotion-focused strategies (managing stress and fear), and avoidance strategies (ignoring abusive comments). |
| Rainey (1994) | To examine the frequency and severity of assaults (verbal and physical) against baseball and softball umpires. | USA | N=782 (M=763; F=19); Sport: Baseball, Softball Age: 43 + 10.8  Experience: 13.6 + 9.2 years (mean and SD) Level: Youth (4%), High school (69%), College/adult (27%) | Quantitative, cross-sectional; Surveys | No theory explicitly stated. Psychological framework identified. | 11% reported being assaulted. No significant differences based on gender or region of the state. Assaults occurred most often during adult/college games. Most common types of assaults were pushing/shoving/grabbing (44%) and Hitting/punching (25%). |
| Rawlings & Anderson (2024) | To examine the experiences of girls and women Australian Football officials | Australia | N=27 (M=0; F=27); Sport: Australian Rules Football | Qualitative; Interviews and focus groups;  Thematic Analysis | Sociocultural framework, specifically, Foucault’s (1979) theory of disciplinary power | Participants experienced sexism and misogyny in interactions with coaches and peers. Participants frequently experienced skepticism and resentment when selected for prestigious matches due to imposter syndrome. Women often self-regulated behavior (e.g., apologized for achievements) to avoid backlash. Racial and gender-based abuse from spectators was also reported. |
| Rayner et al. (2016) | To further explore the concept of abuse within rugby union from a referee’s perspective and evaluate whether the core values of teamwork, respect, enjoyment, discipline and sportsmanship which have historically defined the game, are still part of the sport’s ethos in today’s society. | UK | N=106 (M=0; F=27); Sport: Rugby union  Age: 45-54 (48%), <18, 18-24, 65+ (<10%) | Qualitative; Survey; Content analysis | No theory explicitly stated. Sociocultural framework identified. | 66% of referees surveyed reported experiencing abuse from players, coaches, or supporters. 88.8% of abuse was verbal, 10.1% was physical and 1.2% was mental. The abuse was significant enough for many referees to consider quitting. Although the RFU had formal process for reporting abuse, referees preferred to address the abusers directly. The RFU's reporting system was viewed as complicated, which discouraged officials from using it. |
| Webb et al. (2018) | To examine the extent of verbal and physical abuse, and the training and support offered by referee societies and the Rugby Football League (RFL). | UK | N=89 (M=86; F=3); Sport: Rugby league | Mixed method; Survey; Thematic analysis | No theory explicitly stated. Organizational/ Environmental framework identified. | The findings indicate that 85.4% of referees had experienced verbal abuse, and 16.9% had encountered physical abuse. 20.2% reported verbal abuse in every match, 36% experienced abuse every couple of games, 28.1% faced abuse a few times a season. 44.9% of referees believe abuse had increased in recent years. |
| Webb et al. (2019) | To explore match official perceptions of support and abuse in rugby union and cricket in England. | UK | N=1228 (M=1211; F=17); Sport: Rugby union and cricket Active and non-active match officials included | Mixed method; Survey; Thematic analysis | Sociocultural framework, specifically, Cohen’s (1972) five stages of moral panic. | Results revealed that 49% of rugby union and 45% of cricket match officials experienced abuse at least twice a season, and 51% of rugby union and 47% of cricket match officials believed that abuse had increased. |
| Webb et al. (2020) | To provide an analysis of the experiences of referees in France and the Netherlands related to abuse, conflict, and support in soccer, and to explore potential management resolutions and initiatives to tackle issues concerning abuse, conflict, and support in soccer. | UK | N=4637 (M=4544; F=93) Sport: Soccer | Mixed method; Survey; Thematic analysis | Psychosocial framework, specifically, intergroup conflict theoretical construct | 68.1% of French and 51% Dutch referees reported experiencing verbal abuse. For physical Abuse: France: 16%, Netherlands: 14.6%. Referees felt that reporting abuse was futile due to its frequency and inadequate support. Referees at lower levels report higher incidences of abuse due to unrealistic expectations and closer proximity to spectators. |
| Webb et al. (2017) | To understand how frequently referees experience verbal and physical abuse, and the positive impact of the Respect Program on reducing referee abuse | UK | N=2056 (M=1970; F=72); Sport: Soccer | Mixed method; Survey; Thematic analysis | Sociocultural framework, specifically, figurational sociology theory | 60% of referees reported experiencing verbal abuse at least every few games. Less experienced referees (0-5 years) experienced more frequent verbal abuse. 3.4% of referees with 2 or less years officiating experienced abuse every match. 19% of referees reported physical abuse. Referees questioned the extent to which the Respect Program had succeeded in curbing abuse as they felt respect for them was deteriorating. Referees feel that the sanctions imposed under the Program were not strong enough to deter abusive behavior, further contributing to it. |
